# HTLV-1 infection in acute t- lymphocytic leukemia/lymphoma

**DOI:** 10.4322/acr.2021.307

**Published:** 2021-08-20

**Authors:** Mohammad Barouqa, Morayma Reyes Gil, Radhika Sekhri, Mojisola Popoola, Juan Ding, Yanhua Wang

**Affiliations:** 1 University Hospital of Albert Einstein College of Medicine, Montefiore Medical Center, Department of Pathology, Bronx, NY, USA

**Keywords:** Human T-lymphotropic virus 1, Leukemia, Lymphoid, Leukemia-Lymphoma, Adult T-Cell

## Abstract

Adult T- lymphocyte leukemia/ lymphoma (ATLL), described by Uchiyama et al. in 1977, is a distinct neoplasia of peripheral T-lymphocytes caused by human T-cell lymphotropic virus type 1 (HTLV-1). The authors describe the case of a 75-year-old female patient who presented with fever, chills, and altered mental status. The peripheral blood morphology showed large atypical lymphocytes with multilobed nuclei and flow cytometry consistent with ATLL. The authors discuss the pathophysiology, differential diagnosis, and subtypes of ATLL in addition to the diagnostic approach using flow cytometry when bone marrow biopsy is not available and modalities of treatment.

## INTRODUCTION

ATLL, described by Uchiyama et al. in 1977,^1^ is a distinct neoplasia of peripheral T-lymphocytes caused by human T-cell lymphotropic virus type 1 (HTLV-1). The lack of technological advances in testing at that time limited the detection and any link between HTLV-1 and ATLL. However, Uchiyama et al. suspected a possible viral etiology.^1^ Later on, HTLV-1 was linked to the pathogenesis of ATLL.^2^ HTLV-1 is a retrovirus associated with several autoimmune and inflammatory diseases that include: HTLV-1-associated myelopathy/tropical spastic paraparesis (HAM/TSP),^3^ infective dermatitis associated with HTLV-1 (IDH),^4^ polymyositis, arthropathy, Sjögren's syndrome, and facial nerve paralysis.^5^

Gessain et al.^6^ showed that HTLV-1 varies among geographical regions to include countries like Japan, Africa, Caribbean islands, Central and South America, including Brazil and Peru. It is estimated that there are around 5 to 10 million infected individuals worldwide. There are three modes of transmission for HTLV-1 as follows: (i) Mother to child transmission and mainly linked to prolonged breastfeeding (> 6 months).^7^, (ii) Sexual transmission and mainly from males to females.^8^, and (iii) Transmission through contaminated blood products^9^ and intravenous drug abusers.^10^

The latency period for developing ATLL after HTLV-1 infection can extend from 20 to 30 years, and carriers are at risk of developing ATLL in 6-7% of males and 2-3% in females.^11^

In ATLL, the virus multiplies in the carrier through virological synapse and mitotic division. Infected cells can transmit and transfer the virus, including its RNA, through synapses. The viral RNA is transcribed into DNA in the newly infected cells and later integrated into the DNA to give a new clone. Another mechanism is by the virus, inducing mitotic division of the infected cell resulting in an increase in the number of infected cells. Thus, increasing the number of clones.^12^

## CASE REPORT

A 75-year-old woman with a history of diabetes and hypertension presents to the emergency department with fever and chills. The patient’s symptoms have started gradually two days before her presentation per family with loss of appetite. Upon further reviewing the patient’s history with her family, she has been complaining of episodes of fatigue and muscle cramps recently. Vital signs show hypotension (100/55 mmHg), tachycardia (110 bpm), tachypnea (respiratory rate of 25 per minute), and fever (39.4 C, 102.9 F). Electrocardiogram (ECG) in Emergency Room show sinus tachycardia, and she was admitted to the ICU unit accordingly.

Respiratory examination revealed tachypnea with dullness to percussion over the right side of the chest with decreased air entry over the same area. No wheezing or crackles noted. Furthermore, hepatosplenomegaly was noted on abdominal examination and confirmed by ultrasound later. Her cardiac examination showed only sinus tachycardia. The remaining of her examination is normal. The patient's lab workup is shown in Table 1 and Table 2. Chest X-ray shows a right middle lobe consolidation with mild cardiomegaly. No signs of pneumothorax or pulmonary embolism were seen.

**Table 1:** Hematological workup

Exam	Result	RR	Exam	Result	RR
Hemoglobin	12.8	12.2- 15.3 g/dL	Lymphocyte	73.4	1.0 -4.8k/uL
Hematocrit	39.5	36.0- 45.0%	Monocyte	0.4	0.3-0.5 k/uL
MCV	84.0	80.0- 96.0 fL	Eosinophil	0.3	0.1- 0.3 k/uL
MCH	29.1	27.0 – 33.0 pg	Platelet	272	150- 400 k/uL
MCHC	34.0	33.0- 36.0 g/dL	Iron	79	60- 150 mcg/dL
Leukocytes	80	4.8- 10.8 k/uL	Transferrin	320	300- 360 mcg/dL
Neutrophil	5.9	1.8- 7.7k/uL	Ferritin	190	30- 200 mcg/ L

MCH= mean corpuscular hemoglobin; MCHC= mean corpuscular hemoglobin concentration; MCV= mean corpuscular volume.

**Table 2:** Chemistry workup

Exam	Result	RR	Exam	Result	RR
Sodium	137	135- 145 mEq/L	AG	8	7- 16 mEq/L
Potassium	4.2	3.5- 5.0 mEq/L	EGFR	90	>60 ml/min/BSA
Chloride	103	98-108 mEq/L	Calcium	12.1	8.5- 10.5 mg/dL
BUN		5- 20 mg/dL	Phosphate	2.5	2.5- 4.5 mg/dL
Creatinine	1.08	<1.30 mg/dL	CRP	0.8	<0.81 mg/dL
Glucose	111	70- 140 mg/dL	pH	7.2	7.35- 7.45

AG= anion gap; BSA= body surface area; BUN=Blood urea nitrogen; CRP= C-reactive protein; EGFR= estimated glomerular filtration rate.

The Arterial Blood Gas (ABG) shows hypoxemia and metabolic acidosis.

**Figure 1:**
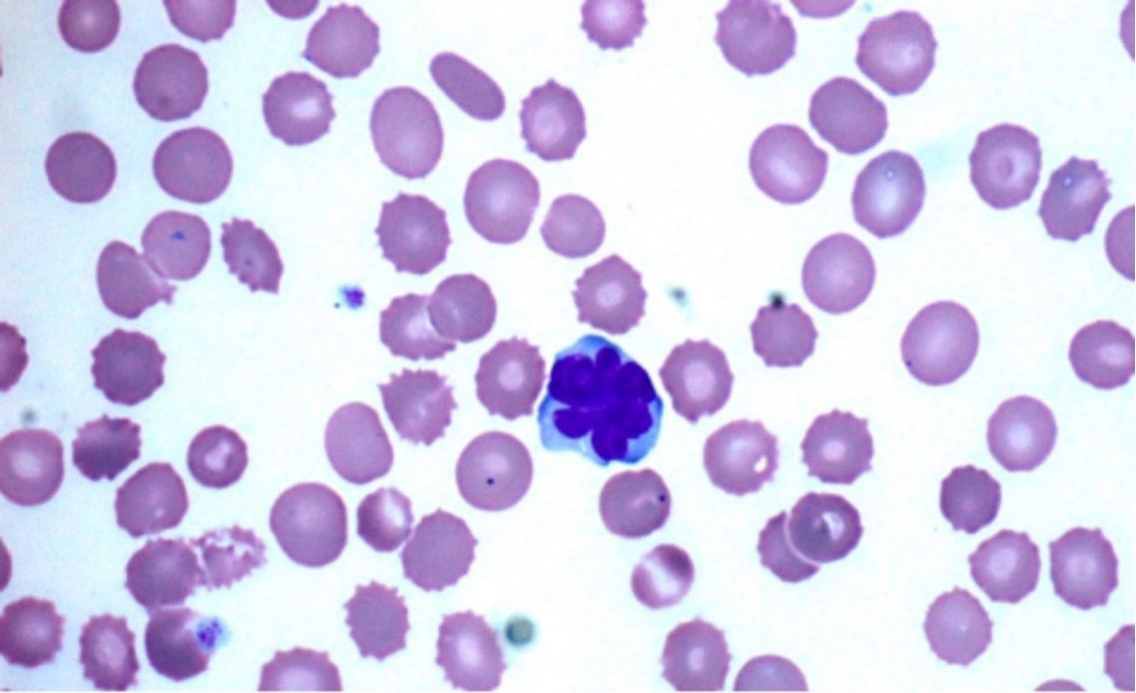
Atypical lymphocytes in peripheral blood smear surrounded by RBC (echinocytes) with small thorny projections. (Original Magnification x1000).

**Figure 2:**
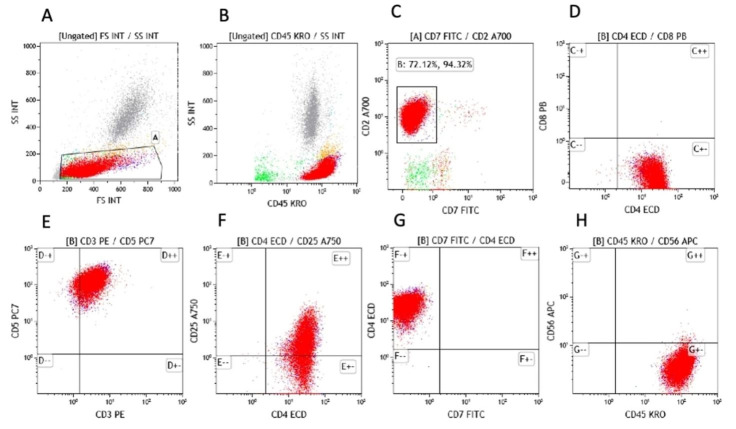
Flow cytometry analysis on peripheral blood lymphocytes. The atypical lymphocytes were detected by blood flow cytometry, gated and painted in red color. A. shows side scatter (SS) versus forward scatter (FS); B. The atypical cells are strongly expressing CD45, C. The atypical cells are expressing CD2, but not CD7, D. The atypical cells are CD4 positive but CD8 negative, E. The majority of atypical cells are expressing CD3 and CD5. F. The atypical cells are expressing CD4 while CD25 Is partially expressed. G. The CD4 positive atypical T-cells are negative for CD7. H. The atypical cells are negative for CD56.

A representative image is pictured from the patient’s blood smear (Figure 1), and based on the morphology findings, flow cytometry was performed. The immunophenotyping of these cells demonstrated mature T lymphocytes with normal pattern expression for the antigens CD45, CD2, CD5 but with weak expression of CD3 and CD25, loss of CD7 antigen, and CD4 T cell subpopulation restriction (CD4+/CD8-). Like the subpopulation T cell CD8, CD56 antigen was not detected as well (Figure 2)

The atypical lymphocytes were detected by blood flow cytometry, gated, and painted in red color. A. shows side scatter (SS) versus forward scatter (FS); B. The atypical cells are strongly expressing CD45; C. The atypical cells are expressing CD2, but not CD7; D. The atypical cells are CD4 positive but CD8 negative; E. The majority of atypical cells are expressing CD3 and CD5. F. The atypical cells are expressing CD4 while CD25 Is expressed partially. G. The CD4 positive atypical T-cells are negative for CD7. H. The atypical cells are negative for CD56.

## DISCUSSION

The patient’s CBC shows a high blood count, which is known as leukocytosis. The differential diagnosis of leukocytosis includes infections, inflammation, leukemia, physical or emotional stress, and post-splenectomy, which is unlikely in the current case since the patient has hepatosplenomegaly. The patient’s pneumonia may explain the leukocytosis, but such significant elevation should raise suspicions of leukemia. However, differential diagnosis of the anemia based solely on the value of Hb, HCT, and MCV includes (i) Iron deficiency anemia, (ii) thalassemia, (iii) sideroblastic anemia, and (iv) myelodysplastic syndrome. Furthermore, the patient's presentation, physical examination, and lab workup are suggestive of acute pneumonia.

The patient’s blood morphology reveals medium to large-sized atypical lymphocytes with multilobulated nuclei resembling flower cells surrounded by echinocytes (Figure.1) without blasts or Rouleaux formation. Echinocytes are red blood cells (RBC) with small thorny projections in the membranes, and it is mainly caused due to improper smear preparation or prolonged sample storage. Nevertheless, they are also seen in end-stage renal diseases and liver abnormalities.

Clinically, the differential diagnosis or initial working up for hypercalcemia should include benign metabolic diseases, such as primary or tertiary hyperparathyroidism, multiple myeloma (MM) with leukemic phase, metastatic malignancy, or neoplasms with paraneoplastic syndrome and lymphomas including ATLL. In conjunction with leukocytosis, hyperparathyroidism is unlikely. MM rarely presents with leukemic phase associated with leukocytosis; the blood smears usually show RBC with Rouleaux formation. However, no RBC Rouleaux formation was seen in this patient’s blood smear. A paraneoplastic syndrome is a group of signs or symptoms related to the neoplastic cells, in which some hormonal-like products are secreted, such as parathyroid hormone-related protein (PTHrP) in small cell carcinoma of the lung. Paraneoplastic syndrome or metastatic carcinoma can be seen with hypercalcemia and leukocytosis, especially at advanced stages. However, the increased leukocytes are usually neutrophils,^13^ instead of atypical lymphocytes. Hence, the diagnosis of carcinoma or paraneoplastic syndrome is less likely either.

The results obtained from flow cytometry and peripheral blood morphology provided deeper insight to the diagnosis.

The flow cytometry analysis demonstrated an aberrant mature T cell population, suggestive of T cell leukemia or lymphoma with blood involvement. The differential diagnosis includes but is not limited to adult T cell leukemia/lymphoma (ATLL), which most commonly express CD4 and CD25 while aberrant loss of CD7 and negative for CD8; Sezary syndrome, or other mature T cell lymphomas, not otherwise specified. To confirm the diagnosis of ATLL, HTLV-1 serology and western blot testing are required.

HTLV-1 serology and western blot testing are also used for confirmation. This Patient’s blood viral serology test is positive for HTLV-1 infection. Together with a positive HTLV-1 testing, the diagnosis is consistent with ATLL.

Flow cytometry is a powerful technology and has been used to identify distinct cell types based on intracellular and extracellular or surface marker expression, an application known as immunophenotyping. It Is an Important test for the diagnosis of ATLL, in which most of the patients displaying a phenotype of mature CD4 T-cells. The following markers are useful in the evaluation of ATLL: CD2, CD3, CD4, CD5, CD7, CD8, CD25, CD26, CD45RO, and αβ T-cell receptors. Many cases of ATLL do not express CD7 and CD26, but they can show decreased expression of CD3.^14^^,^ 15

Table 3 summarizes key distinguishing characteristics of other T- cell neoplasms from ATLL.^16^^,^ 17

**Table3:** Types of T- Cell Leukemia/ Lymphoma and their characteristics

**Type of T- Cell leukemia/ lymphoma**	**Characteristics**
Peripheral T- cell leukemia/ lymphoma, not otherwise specified (PTCL- NOS)	Negative serological and molecular tests for HTLV-1.
	Effaced lymph nodes with reactive plasma cells, eosinophils and histiocytes in the background.
	Predominant in the western hemisphere.
Angioimmunoblastic T- cell lymphoma	Atypical lymphoma cells positive for CD10, Bcl-6, PD-1 and CXCL13, and IDH2 R172 mutation.
	Prominent proliferation of CD21 follicular dendritic cells in affected lymph nodes.
	Hypergammaglobinemia and skin rash.
T- lymphoblastic leukemia/ lymphoma	Large mediastinal masses in young patients
	TDT and CD1a positive.
Mycosis Fungoides/ Sezary syndrome	Sezary syndrome presents with small atypical T cells and cerebriform and hyperchromatic nuclei.
	History of skin disease with intraepidermal Pautrier’s microabscesses.
	Atypical T cells usually do not express CD25.
Anaplastic large cell lymphoma	Lymph nodes with hallmark cells, positive for CD30, and another cytotoxic T cell phenotype.

The clinical diagnosis of ATLL is based on detecting the seropositivity for HTLV-1 in patients, along with hematological and hematopathological diagnosis of peripheral T-cell leukemia/ lymphoma.^14^ ELISA test is generally the first initial test to detect HTLV-1 in serum, followed by Western Blot and/or PCR for confirmation. In peripheral blood lymphocyte morphology, the presence of “flower cells” is highly pathognomonic of ATLL. These flower cells are medium to large atypical lymphocytes with multilobed nuclei with dense chromatin and small/absent nucleoli.^18^ These flower cells carry the antigens tested by flow cytometry.

ATLL is clinically subcategorized into four main subgroups: acute, chronic, lymphomatous, and smoldering, as shown in Table 4.

**Table 4:** Clinical forms of ATLL

**Clinical manifestation**	**Acute**	**Chronic**	**Lymphomatous**	**Smoldering**
Number of lymphocytes	Increased	Mildly increased	Normal	Normal
Abnormal lymphocytes in peripheral blood	Increased	Mildly increased	Initially absent	> 5%
Hypercalcemia	Present	Absent	Variable	Absent
Lymphadenopathy	Usually present	Mild	Present	Absent
Hepatosplenomegaly	Usually present	Mild	Usually present	Absent
Bone marrow infiltration	Often present	Absent	Absent	Absent
Median survival	< 1 year	> 2 years	< 1 year	> 2 years

The prognosis of ATLL in patients with acute or lymphomatous subtypes is generally poor, with a median overall survival measured in months, mainly less than 1 year. The tumor cells in these variants have an intrinsic resistance to most chemotherapeutic agents, and because the patients have an underlying immunocompromised state associated with their HTLV-1 infection, treatment can be indeed a challenge. Poor prognosis in these patients can be explained by the impaired cell-mediated immunity while humoral immunity remains intact.

The main cornerstone for treating these patients is to use combination chemotherapy after an intensive initiation phase. The defucosylated humanized anti-CCR4 antibody mogamulizumab appears to improve response rates and progression-free survival, but is not widely available outside of Japan, and it is associated with an increased risk of transplant complications. Autologous hematopoietic cell transplantation (HCT) does not appear to be effective. Allogeneic HCT may be appropriate for patients with a related or unrelated donor and may result in long-term disease control. There are several trials that investigated the effectiveness of different regimens of chemotherapy. The regimen that appears to result in the longest median survival is VCAP-AMP-VECP (also known as LSG15), which includes treatment with vincristine, cyclophosphamide, doxorubicin, prednisone, ranimustine, vindesine, etoposide, and carboplatin.^19^

The benefit of using antiviral therapy is still controversial. The HTLV-1 virus is thought to be in a latent state in patients with ATLL. Hence, if any benefit to be proven for the use of such medications, it will be through different mechanisms other than the antiviral pathway.^20^

HTLV-1-seropositive individuals should be advised not to donate blood, semen, organs, or milk, where milk banks are available. Prevention of mother-to-child transmission would probably have the most significant impact on the occurrence of HTLV-1 infection and associated diseases.^21^

Prenatal screening for HTLV-1 should be implemented in specific geographical areas, combined with counseling of seropositive mothers regarding transmission through breastfeeding. Avoidance of breastfeeding is fundamental, since it is the major form of vertical transmission of HTLV-1.^21^

Due to the risk of malnutrition in developing countries, public health policymakers should consider this adverse effect in less developed countries and recommend an alternative feeding formula for children at risk of acquiring HTLV-1 infection through mother's milk.^19^ Recommendations to prevent sexually transmitted infections should be routinely counseled, including condom use and avoiding multiple and unknown sexual partners. When one of the partners in a stable relationship is negative, the need for mechanical barriers use should be emphasized.

Counseling and education of intravenous drug users (IDU) to implement harm reduction practices may be effective in preventing HTLV-1 infection in this population group.

## CONCLUSIONS

HTLV-1 infection plays a pivotal role in ATLL. We described the clinical presentation of ATLL and highlighted the pathophysiology, importance of peripheral blood smear, and flow cytometry in the diagnosis and management of this entity.
